# Production, Preservation, and Transfer of South American Camelid Embryos

**DOI:** 10.3389/fvets.2017.00190

**Published:** 2017-11-13

**Authors:** Virginia L. Trasorras, María Ignacia Carretero, Deborah M. Neild, Maria Graciela Chaves, Susana M. Giuliano, Marcelo H. Miragaya

**Affiliations:** ^1^Facultad de Ciencias Veterinarias (FCV), Instituto de Investigación y Tecnología en Reproducción Animal (INITRA), Cátedra de Teriogenología, Universidad de Buenos Aires (UBA), Buenos Aires, Argentina; ^2^Consejo Nacional de Investigaciones Científicas y Técnicas (CONICET), Buenos Aires, Argentina; ^3^Facultad de Ciencias Veterinarias (FCV), Instituto de Investigación y Tecnología en Reproducción Animal (INITRA), Cátedra de Física Biológica, Universidad de Buenos Aires (UBA), Buenos Aires, Argentina

**Keywords:** camelids, embryo, IVP, semen, reproductive biotechnologies

## Abstract

The current review summarizes progress in the field of *in vitro* and *in vivo* production of South American Camelid embryos. Both methods require ovarian superstimulation (with FSH and eCG) to obtain multiple ovulations (*in vivo* embryo production) or to induce follicle growth for oocyte collection (*in vitro* embryo production). Moreover, superstimulation entails prior administration of hormones that inhibit follicular growth (progesterone, progestagens, and estrogens). Cumulus-oocyte complexes obtained must mature *in vivo* (buserelin administration) or *in vitro* to then be subjected to *in vitro* fertilization or intracytoplasmic sperm injection. All these techniques also require morphologically normal, motile spermatozoa to achieve fertilization. Methods used to decrease semen viscosity and to select the best spermatozoa (Percoll^®^; Androcoll-E^TM^) are described. Additionally, nuclear transfer or cloning has been applied in llamas. Up to now, embryo deep-freezing and vitrification have progressed slowly but are at the height of development. Embryos that are obtained by any of these techniques, either *in vivo* or *in vitro*, need to be transferred to synchronized recipient females. The best results are achieved after transfer to the left uterine horn with an ipsilateral ovulation. No live offspring have been obtained after the transfer of cryopreserved embryos. Applying reproductive biotechnologies, such as those described, will permit the expansion of genetically selected animals in the population and also that of wild camelid species, vicunas, and guanacos, whose embryos could then be transferred to the uterus of domestic species.

## Introduction

*In vitro* and *in vivo* embryo production is extensively used in domestic species such as bovines and equines. The industry that surrounds these species promotes the research and development of these biotechnologies, but in South American Camelids (SAC), reproduction-focused research has been minimal. This is mainly due to multiple unique reproductive characteristics such as: induced ovulation with a short half-life of the corpus luteum (CL) (1) combined with different luteolytic activity between uterine horns (2), added to which the period for maternal recognition of pregnancy (MRP) is relatively short (3) and pregnancies only establish in the left uterine horn (4, 5); males present low sperm concentration in their ejaculates (6, 7), which are highly viscous and show thread formation (8), all of which compound to make research somewhat complex. Nonetheless, progress has been achieved in some assisted reproductive techniques (ART), such as synchronization of ovarian follicular development, ovarian superstimulation, and embryo transfer (ET), while others present fewer advances, for example, artificial insemination (AI), especially when using cryopreserved semen, *in vitro* fertilization (IVF) and intracytoplasmic sperm injection (ICSI). In addition, crucial reproductive physiology, such as MRP signaling, remains in parts unknown.

Applying biotechnology, such as *in vitro* and *in vivo* embryo production, ET and gamete cryopreservation, offers the possibility of increasing knowledge on embryo and gamete requirements, favors genetic progress, and makes future commercialization possible.

This review discusses some of the reproductive techniques necessary for embryo production that are available today in SAC and that can be applied in genetically superior females and males.

## *In Vitro* Embryo Production

*In vitro* production of embryos requires the provision of large numbers of competent oocytes apt for fertilization. This gamete can be collected from dead or live animals. The methods used to obtain cumulus-oocyte complexes (COCs) are: aspiration or dissection of follicles from slaughterhouse ovaries (9–12); aspiration of follicles surgically exposed during a laparotomy [llamas (13–16); alpacas (17)] or ultrasound-guided transvaginal aspiration of follicles (18–21).

### Postmortem Oocyte Collection

This technique is the most often used for oocyte collection, especially in South American countries like Peru or Bolivia, because they have abattoirs especially dedicated to camelids. The highest yield of oocytes (27 per llama) can be obtained from slaughterhouse ovaries, but their degree of maturation is low (9). The large numbers of oocytes provided is very advantageous, but the main drawback is that *in vitro* maturation (IVM) needs to be carried out before they can be used for IVF (22). Complete maturation requires both nuclear and cytoplasmic maturation and “from a review of the literature, it is evident that maturation procedures for llama oocytes are not optimal” (22). Del Campo et al. published the first report of IVM of llama oocytes in 1992. Since then, based on oocytes reaching the metaphase II stage (MII) of development, most researchers have reported 39–80% nuclear maturation of oocytes between 24 and 36 h [llamas (11, 23); alpacas (12)]. Evaluation of cytoplasmic maturation, however, has not been reported for SAC oocytes coming from slaughterhouse ovaries. Nonetheless, there does appear to be a consensus that llama oocytes from slaughterhouse ovaries need approximately 36 h in culture to mature to the MII stage (11, 23), and in the case of alpacas, 32 h resulted in the highest rate of oocytes reaching the MII stage (12). Furthermore, as oocytes were recovered from an abattoir, it is unknown if the dominant follicles were growing or regressing, a fact that would affect oocyte quality (24).

Thus, this method of oocyte collection has the advantage that a lot of gametes can be obtained from a single ovary, but also has the disadvantage of needing *in vitro* oocyte maturation, and this procedure is not yet set up. More research is indispensable to increase knowledge regarding oocyte requirements for IVM.

### *In Vivo* Oocyte Collection

Obtaining oocytes from live animals offers the possibility of increasing the offspring of genetically superior females and decreasing the generation gap. SAC species are monotocous and as such, various follicles are recruited at the beginning of each follicular growth phase, but only one follicle, the dominant one, will reach an ovulatory diameter of 7 mm (25). Due to this, to obtain various follicles from a female, it is necessary to previously apply ovarian superstimulatory treatments.

#### Control of Ovarian Dynamics

Follicular waves in oocyte donor females must be evaluated daily using transrectal palpation and ultrasonography. In llamas, ovarian superstimulatory treatment should be initiated when no dominant follicle is present in the ovary (26) and when commencing treatment in the presence of follicles larger than 5 mm, excessive development of only one follicle will take place (27).

Due to these findings, superstimulation treatments are applied to donor females in the absence of follicles greater than 5 mm (controlled by ultrasound). Various protocols have been developed to inhibit ovarian dynamics based on the negative effect progesterone has on follicular activity in the presence of a CL (3). A natural luteal phase (inducing ovulation) or an artificial one (applying progesterone or exogenous progestagens) can be used. These can be found as injections (short or long-acting progestagens) or as releasing devices: subcutaneous implants with progesterone (Norgestomet^®^, 3 mg), intravaginal devices (CIDR^®^, 0.33 g; used in small ruminants), or intravaginal sponges (medroxyprogesterone acetate, different concentrations). They can also be combined with injectable estrogens (17 β estradiol, estradiol benzoate, estradiol valerate). In llamas, it has been reported that the progesterone from the intravaginal device (0.33 g) inhibits ovarian activity, despite follicle development at the moment of insertion (28), indicating that the treatment can be initiated without prior examination of the ovary. The CIDR^®^ is a practical and simple method for use in the field, which enables inhibition of follicular waves in llamas between days 5 and 8 after insertion, at which time, the ovaries can receive superstimulatory treatment with gonadotrophins as there is no dominant follicle present (29). The problem with this device is that it is no longer available on the market in our country; therefore, we need to resort to new alternatives. Alberio and Aller (30) used 50 mg of injectable progesterone over a period of 12 days, obtaining a follicle diameter below 5 mm on day 7. Carretero et al. (31) evaluated the effectiveness of administering 100 and 150 mg of progesterone daily for 5 days and as soon as day 3, follicles decreased their size to 5 mm. These protocols are highly effective in llamas, but their disadvantage is they are not practical for use in the field (daily injections are needed) and produce pain at the site of injection. For these reasons, it is useful to evaluate the effect of long-acting progesterone (requiring only one injection; BioRelease^®^ LA 300, BETPharm) on ovarian dynamics in the llama. The formulation is prepared to release progesterone for approximately 10–12 days after intramuscular administration. In horses, it has been proven that 5 ml (1,500 mg) of this compound maintains high progesterone levels (>4 ng/ml) for 10 days (32). Our group has started to evaluate its effect in llamas.

#### Ovarian Superstimulation

The drugs most used in camelids to induce ovarian superstimulation are FSH and eCG, either individually or combined. According to Bravo et al. (33) administration of 500 or 1,000 IU of eCG, in absence of dominant follicles (controlled by ultrasound) are the optimal doses for superstimulation in llamas; a rise in the occurrence of cystic follicles was observed with higher doses (2,000 IU). However, 500 IU of eCG did not produce ovarian superstimulation after follicle inhibition with estradiol benzoate and CIDR^®^ in this species (14). According to our experience, treatments with 1,000 or 1,500 IU of eCG are effective for inducing multiple follicle growth, but a larger number of follicles are obtained with 1,500 IU (14, 31). Administration of high doses of eCG can be beneficial for carrying out follicle aspiration to obtain COCs, but when the objective is to obtain embryos through uterine flushing, it could be detrimental due to the displacement of the ovarian bursa because of the large size of the superstimulated ovary. In vicuna, administration of a dose of 750 IU of eCG produced a potent stimulation of follicle growth, obtaining an average of 16.5 follicles per female (34). According to our experience, a problem with the use of this hormone is that some llamas tend to become refractory to eCG following multiple administration. This suggests that at least in the llama, the risk of inducing anti-eCG antibodies is real.

#### Oocyte Recovery

The technique with the highest percentages of oocyte recovery in llamas and alpacas is follicular aspiration by laparotomy (Table 1). With this method, over 80% of the follicles yield COCs [llama (14); alpaca (35)], and we observed in the llama that the stage of maturation COCs had attained was related to the size of the follicle that was aspirated (14). Chaves et al. (36) obtained a 55.4% recovery rate in vicunas (46 oocytes recovered from 83 aspirated follicles). However, this method requires both a team of specialists in surgery, anesthesia, and an operating theater. This surgical technique is also very invasive, therefore, ovum pick-up using the ultrasound-guided transvaginal method simplifies oocyte recovery while at the same time is an *in vivo* method that is quick and non-traumatic. The limited bibliography available in superstimulated llamas on this technique reports a COCs recovery rate of 52–77% (19–21). As a disadvantage to both the laparotomy and ultrasound-guided transvaginal follicle aspiration techniques, one should mention the possibility of bleeding after the aspiration and, therefore, due to the release of fibrin, adherences can be formed in the ovarian bursa (24).

**Table 1:** Different treatments applied for *in vivo* oocyte’s recovery in South American Camelids.

Species	Follicular inhibition treatment/control	Superstimulatory treatment	Method of oocyte collection	Recovery rate (no. oocytes collected/follicle aspirated; *n* = females)	Reference
Llama	Not specified	400 mg FSH + 100 IU eCG	TUGA	52% (45/86; *n* = 5)	Brogliatti et al. (19)
Llama	CIDR	500 IU eCG + FSHp (doses not specified)	Laparotomy and needle aspiration	59% (39/66; *n* = not specified)	Miragaya et al. (13)

Llama	TUGA	200 mg FSH	TUGA	73% (not specified; *n* = 18)	Ratto et al. (20)
		1,000 IU eCG		74% (not specified; *n* = 17)	

Alpaca	Follicular dynamic control by ultrasonography	200 mg FSH	Laparotomy and needle aspiration	83% (not specified; *n* = 4)	Gomez et al. (35)
		1,200 IU eCG		82% (not specified; *n* = 7)	

Vicuna	Follicular dynamic control by ultrasonography	750 IU eCG	Laparotomy and needle aspiration	55.4% (46/83; *n* = 4)	Chaves et al. (36)

Llama	Manual rupture of dominant follicle	200 mg FSH	Ovariectomy	97% (298/307; *n* = 9)	Sansinena et al. (37)

Llama	TUGA	200 mg FSH	TUGA	71% (193/273; *n* = 18)	Ratto et al. (11)
		1,000 IU eCG		74% (192/258; *n* = 17)	

Alpaca	Follicular dynamic control by ultrasonography	200 mg FSH	Laparotomy and needle aspiration	89% (105/118; *n* = 4)	Ratto et al. (17)
		1,200 IU eCG		87% (163/187; *n* = 7)	

Llama	Manual rupture of dominant follicle	7.92 mg ovine FSH	TUGA	61.5% (16/26; *n* = 4)	Sansinena et al. (38)
			Laparotomy and needle aspiration	94% (128/136; *n* = 7)	

Llama	CIDR + 1 mg estradiol benzoate	1,000 IU eCG	Laparotomy and needle aspiration	86.5% (193/223; *n* = 21)	Conde et al. (39)

Llama	CIDR + 1 mg estradiol benzoate	1,000 IU eCG	Laparotomy and needle aspiration	83.1% (69/83; *n* = 10)	Trasorras et al. (14)
		1,500 IU eCG		81.7% (112/137; *n* = 9)	

Llama	TUGA	200 mg FSH	TUGA	77% (185/240; *n* = 16)	Berland et al. (21)
		1,000 IU eCG		71.5% (156/218; *n* = 16)	

Llama	Follicular dynamic control by ultrasonography	1,500 IU eCG	Laparotomy and needle aspiration	87% (69/79; *n* = 12)	Trasorras et al. (22)

Llama	Follicular dynamic control by ultrasonography	1,500 IU eCG	Laparotomy and needle aspiration	86% (66/77; *n* = 11)	Trasorras et al. (15)

*TUGA, transvaginal ultrasound-guided oocyte aspiration*.

#### *In Vivo* Oocyte Maturation

As already mentioned, in SAC, the rate of *in vitro* ooctye maturation is still variable. Taking into account that induced ovulation is a characteristic of these species; *in vivo* oocyte maturation within the follicles could be produced by inducing an LH surge. This observation is supported by reports in bovines, where more embryos reach the blastocyst stage after *in vivo* maturation than after IVM (40, 41). Meiosis start-up can be induced by administration of buserelin or hCG, both of which are gonadotrophin releasing hormone (GnRH) analogs. In llamas, optimal time for surgical oocyte recovery has been reported as 20 h after buserelin administration (14). A larger proportion of expanded COCs and COCs in metaphase II were obtained after superstimulation with eCG compared to treatment with FSH (11). Trasorras et al. (14) reported the beneficial effect of administering buserelin in llamas, as a larger number of expanded COCs were recovered and proposed that this protocol could be used directly in ART without needing prior maturation. In this same study, these authors aspirated follicles ≥7 mm in diameter in females supestimulated with eCG, obtaining 94% (98/104) COCs in the expanded stage whereas no compact COCs (0/104) were recovered (14). In vicunas, Chaves et al. (36) superstimulated females with 750 IU of eCG and studied oocyte nuclear and cytoplasmic maturation. Oocytes were fixed immediately after surgical aspiration and evaluation was carried out by staining nuclear material with propidium iodide (PI) and the cortical granules with fluorescein marked peanut agglutinin (FICT-PNA) and then analyzing them with confocal laser microscopy. The results showed a 45% nuclear maturation but only 9% of the oocytes also evidenced cytoplasmic maturation in the controls; whereas superstimulated females evidenced 41% nuclear and cytoplasmic maturation, demonstrating that although ovulation was not induced, superstimulatory treatment exerted a beneficial effect on oocyte maturation (24).

Hence, *in vivo* oocyte, collection is more laborious than *in vitro* oocyte collection. If the LH surge of the female is not induced, *in vitro* oocyte maturation will be needed in both recovery procedures. The source of the female gamete will depend on our possibilities and the animals that are available at the time.

### *In Vitro* Embryo Production Techniques

#### IVF and ICSI

**Figure 1:**
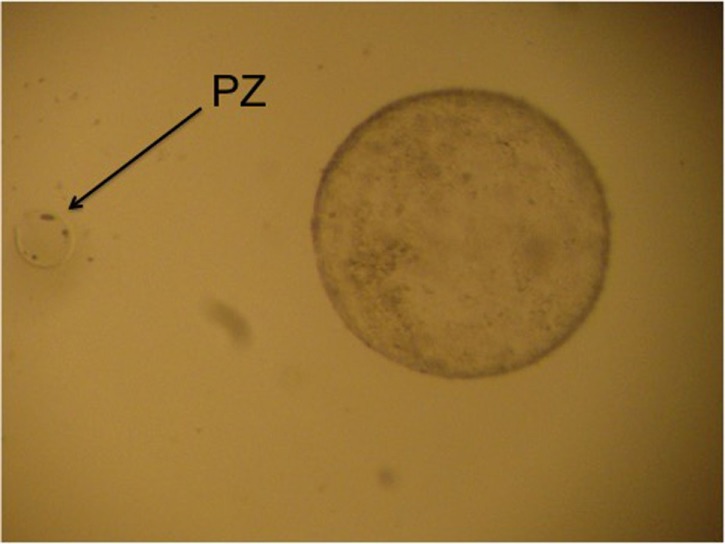
“Hatched llama blastocyst produced on the sixth day of culture in SOFaa medium renewing every 48 h and semen processed with Androcoll-E™. PZ, pellucid zone.”

Among the few articles published on IVF in camelids, in the first publication on this subject, Del Campo et al. (9) used coculture with oviduct cells, abattoir llama oocytes, and sperm from the epididymus, obtaining only 11 hatched blastocysts from 234 cultured zygotes (4.7%). Later, Gomez et al. (35) employed coculture with bovine oviduct cells for heterologous IVF, using llama epididymal sperm and a small number (*n* = 5) of slaughterhouse alpaca oocytes to produce the first llama–alpaca crossbreed embryos. Although all became morula after 6 days of *in vitro* culture, development stopped at that stage. Our group has employed IVF (15, 16, 39, 42) and ICSI (39, 43) to produce llama embryos *in vitro*. The study by Conde et al. (39) represents preliminary development of IVF and ICSI using raw llama semen collected using electroejaculation, obtaining embryos produced *in vitro* that developed to the expanded blastocyst stage for the first time. Furthermore, the first report of a pregnancy in llama was by Trasorras et al. (15) after the intrauterine transfer of embryos produced by IVF using fresh semen processed through Androcoll-E™ and oocytes obtained from superstimulated females (Figure 1). So far, the birth of only one llama (1/6) with IVF has been reported using slaughterhouse gametes (44). However, to date, the results obtained regarding early embryo development are poor, mainly because the quality of the blastocysts produced *in vitro* is not as good as that of blastocysts produced *in vivo*. Supporting this observation, various studies found that the number of blastomeres in pig blastocysts produced *in vitro* was lower than in those produced *in vivo*, despite not differing in number between the different *in vitro* culture media used (45–47). Increased reproductive efficiency of both domestic and wild camelids could be achieved with the use of this kind of assisted reproductive technique in genetically superior animals, as an important increase in the number of embryos produced *in vitro* could be attained as a result of their implementation (22). This biotechnology is also fundamental to reproduce females and males with some specific acquired reproductive disorders preventing fertilization or pregnancy maintenance.

#### Cloning

Nuclear transfer or cloning is a technique that is mainly used in animals of high genetic value or whose production justifies the application of this procedure. This is because of its high economic cost, not only due to the need for a high number of oocytes but also because of the need to have a micromanipulator and highly trained personnel. The genetic material of the animal that is to be cloned is inserted into the cytoplasm of an oocyte that has had its nucleus removed. There are no reports of the application of this technique in alpacas and there is only one report in llamas. In 2003, Sansinena et al. created 80 couplets using oocytes obtained from superstimulated and ovariectomized llama females, which were then *in vitro* matured prior to receiving a male llama fibroblast cell obtained from a skin biopsy. They achieved a success rate of 62.5% fusions with cleavage rates ranging between 32 and 40%. However, no pregnancies were detected on day 14 after transferring a total of 11 embryos (stage 8 cells to morula) to synchronized recipient llamas. Conversely, Wani et al. (48) were successful in obtaining the first live clone in dromedary camels from a reconstructed embryo using cumulus cells.

### *In Vitro* Culture of Embryos

Possible *in vitro* embryo culture techniques include: (1) coculture with diverse cell types such as oviduct epithelial cells (9, 49–51), granulosa cells (51, 52), and somatic cells (53) or (2) employing synthetic culture media, either defined or semi-defined. Among the latter, the following should be mentioned: synthetic oviductal fluid (SOF) (54–56), Charles Rosenkrans (CR) media 1 (57), CR 2 media (58), potassium simplex optimization medium (KSOM) (59), and Dulbecco’s modified Eagle’s Medium (DMEM-F12) (60). However, the use of an undefined medium makes it complicated or nearly impossible to analyze embryo nutritional requirements and, in the case of cell coculture, adds to the variability in culture system composition (61). In addition, competition for the nutrients present in the medium could occur between the embryos and the coculture cells and cell metabolic waste could also negatively impact embryo development (24). The designing of defined culture media is based on the studies in embryo dynamics (both physiological and metabolical) and on data recovered from the oviduct microenvironment (24). In SAC, there are few results on the composition of the fluid produced by the oviduct [alpacas: (62); llamas: (63)]. Compared to other species, early *in vivo* embryo development in llamas seems to be fast as it has been possible to recover morula-stage embryos *in vivo* from llama oviducts as soon as 3 days after ovulation (64). It is to be expected that adequate nutrients, ions, hormones, proteins, amino acids, and growth factors are indispensable components of the oviductal microenvironment for this rapid growth to be possible; therefore, to be able to improve the current culture media, it becomes essential to acquire information on the relative quantities present in the oviducts of these species.

Not only is the composition of the culture media important for the development of an *in vitro* embryo culture system, but another vital factor to consider is the amount of time the embryos are in contact with this media. Furthermore, the embryos also contribute to the culture medium they are placed in, especially when amino acids are added and, therefore, the culture system should not be regarded as static (15). “Studies carried out in humans (65), mice (66), and sheep (67) for *in vitro* culture embryo development to the blastocyst stage, have demonstrated that although the addition of amino acids to the culture medium had a significant effect on both embryo cleavage rate and morphological development, the beneficial effects on cleavage decreased in relation to the duration of culture” (15). The presence of aminoacids in the culture media produces an increase in the concentration of ammonium due to their spontaneous breakdown at 37°C, which is added to that produced by the growing embryo’s metabolism (67). As a result, this increase in ammonium inhibits embryo cell division and the development of the blastocyst [human: (65); mouse: (66)]. In addition, in mice, it has been associated with a delay in fetal growth and neural tube defects (68). With the aim of alleviating the toxic effects of ammonium, sheep embryos were placed in fresh media every 48 h, resulting in increased embryo cleavage and development rates when in culture (67).

By contrast, in llamas in the study by Trasorras et al. (15), when the culture medium was renewed every 48 h for 6 days, the hatching blastocyst rate was lower than that obtained using the same culture medium without renewal (58 vs. 80%, respectively). However, the renewal of the culture medium allowed a swift growth of embryos, as was evidenced on day 4 of culture by the presence of hatched blastocysts. Khatir et al. (51) reported a similar rapid growth 5 days post-IVF in dromedary *in vitro* embryo production using coculture with somatic cells; however, in other domestic species, such as bovines, blastocysts are usually observed on day 6–7 of *in vitro* culture (61). “In this species, transcrips and proteins from the oocyte govern initial embryonic development after fertilization until the fourth cell cycle and, at this stage, the embryonic genome control of development becomes evident (69). It is possible that in camelids, the embryonic genome controls the development of the embryo earlier than in the bovine species” (15).

### Semen Preparation

Although important contributions to the knowledge on SAC’s reproductive physiology and advances in the development and application of biotechnology have been made in the last few years, it is crucial to improve the current methods of sperm selection of these species, with a special emphasis on their seminal characteristics, and more studies are required to attain this objective.

#### Semen Characteristics

It is important to consider that SAC semen has some very particular characteristics. Among them, one has to mention the low volume and low sperm concentration, both of which limit the number of assays that can be carried out on each ejaculate [llamas: (6, 70, 71); alpacas: (7)]. In addition, sperm in raw SAC ejaculates that have not been enzymatically treated to reduce thread formation show an oscillatory pattern of movement [llamas: (6, 71, 72); alpacas: (7)]. Related to this characteristic of thread formation is the high structural viscosity that was reported by Casaretto et al. in 2012 in llamas and which should be taken into account. Furthermore, high variability for the different seminal parameters is observed both between llama males and even between ejaculates from the same male (70). Contrary to other species, such as humans for example, semen liquefaction does not occur either after incubation at 37°C or after being left at room temperature [llamas: (73)] and in the cases, it does occur, it takes too much time (24 and 48 h) [alpacas: (74)], thus making it impossible to use that ejaculate. Both the abovementioned structural viscosity and thread formation make it very difficult to manage ejaculates and to separate both spermatozoa from seminal plasma and separate sperm according to their motility patterns (38, 39, 72, 75). Due to these characteristics, Del Campo et al. (9) highlighted the fact that they obtain llama embryos using sperm from the epididymis and not ejaculated sperm. Likewise, Sansinena et al. (38) discarded the use of IVF in llamas because of the low percentages of spermatozoa with progressive motility.

Currently, ART in SAC are used in research laboratories. For this reason, the gametes used belong to a wide range of animals, independently of their quality. However, the objective of commercial application of these ART is to use them in animals of high genetic value. Sometimes genetically superior animals are not those with the best seminal quality.

“Therefore, semen processing techniques must be capable of separating the higher quality spermatozoa, even from samples recovered postmortem” (76).

#### Separation of Spermatozoa from Seminal Plasma

“Enzymes have been used to improve rheological characteristics of SAC seminal plasma, with varying results” (24). Bravo et al. (77) incubated alpaca ejaculates in aqueous solutions of tripsin and collagenase. Although these authors observed a decrease in viscosity, they also observed a decrease in the percentage of sperm with progressive motility. In another study, Bravo et al. (78) found that the enzymes trypsin, collagenase, hyaluronidase, and fibrinolysin were effective for decreasing alpaca and llama semen viscosity, but they were unable to obtain sperm with progressive motility. Using tripsin in the media, Poblete et al. (79) observed a high percentage of detached sperm heads in raw llama and alpaca semen. Similarly, Maxwell et al. (80) observed that collagenase was toxic for alpaca spermatozoa at all concentrations tested (0.5, 1.0, 2.0, and 4.0 mg/ml). With the objective of improving seminal characteristics, our laboratory has implemented a protocol that decreases llama ejaculate viscosity, separates sperm from seminal plasma, and induces progressive motility. In this protocol, 0.1% collagenase (1 mg/ml of HEPES-TALP-BSA medium) is added to the ejaculate at a dilution of 1:4, incubated 4 min at 37°C, and then centrifuged for 8 min at 800 *g* and the pellet is re-suspended in 2 ml HEPES-TALP-BSA (72). Using this protocol, absence of thread formation was observed, increasing the average percentage of progressive motility (from 4.4 to 41%) and greatly decreasing the percentage of sperm oscillatory motility (30.5 to 7–9%). In addition, comparison of the samples incubated with collagenase to the raw ejaculate showed no significant differences (*p* > 0.05) in the proportion of viable sperm whereas greater percentages (*p* < 0.05) of cells with functional membranes were obtained after collagenase incubation (72). This same study reported similar results with a slightly modified version of the protocol, where incubation at 37°C is increased to 8 min if samples are extended 1:8 in the same solution of 0.1% collagenase. In another study in alpacas, Kershaw-Young et al. (81) evaluated the effect of glycosaminoglycan enzymes (hyaluronidase, chondroitinase ABC, and keratinase) and proteases (papain and proteinase K) on ejaculated sperm, in order to propose alternative methods to reduce semen viscosity. In this study, papain proved to be the most effective enzyme for eliminating thread formation, achieving this effect within 30 min. Although all enzymes accomplished the desired effect of significantly diminishing seminal plasma viscosity (*p* < 0.001) without affecting either sperm motility or DNA integrity, the percentages of intact acrosomes and viable sperm were reduced in all enzyme-treated samples with the sole exception of papain (*p* < 0.001). Recently, these same authors reported that semen viscosity can be eliminated in alpacas using a 20 min incubation at 37°C with papain (0.1 mg ml/1) and that this treatment does not reduce the percentages of viable, motile sperm, or those with intact DNA and acrosomes (82). Studies are necessary to determine if papain is equally effective to reduce thread formation of llama semen.

#### Selection of Motile Spermatozoa

Gamete quality (both of the female and the male gametes) is of vital importance when ART are applied. Thus, it is imperative to be able to select normal, functional sperm, and separate them from cell debris and the dead spermatozoa also present in an ejaculate (76). Accordingly, any method used to separate motile sperm should not damage cells in any way (either morphologically or physiologically) (83). The requirements for these techniques are simplicity, speed, low cost, and the capacity to remove non-spermatic cells, dead sperm, toxic substances, decapacitating factors, and factors producing reactive oxygen species (ROS).

The two main methods of sperm selection are the swim-up technique and density gradient centrifugation. The swim-up technique was developed by Parrish and Foote (84) and is the one most commonly used in human medicine (83). In this procedure, a support medium is layered on top of the ejaculate and selection is based on the capacity of motile spermatozoa to swim away from seminal plasma and up to the overlaying media (76). This technique has the advantage of being simple to perform inexpensive and that a fraction rich in morphologically normal motile spermatozoa can be obtained. Nevertheless, it has the disadvantage of recovering a low number of llama spermatozoa (85) and so needs to be used in ejaculates with a high sperm count and motility. Furthermore, spermatozoa can be greatly damaged by ROS during incubation (86). In llamas, there is only one report that evaluated the swim-up technique using semen obtained by electroejaculation and layering either the whole ejaculate (with seminal plasma) or semen samples after removal of seminal plasma with collagenase (85). In this study, ejaculates were incubated with collagenase (1 mg/ml) to decrease thread formation and then half was centrifuged and re-suspended in a medium before being layered over the swim-up culture medium (without seminal plasma). The other half was submitted to swim-up without prior centrifugation (with seminal plasma and collagenase). No significant differences were observed in the percentage of endosmosis, viability, and progressive and total motility of recovered sperm between the two swim-up protocols. However, very low concentration and total sperm were obtained with both methodologies. These results confirmed that swim-up is not an adequate method of sperm selection in llamas, even after collagenase treatment. However, this technique gave acceptable results when used on alpaca epididymal sperm (12). These authors did not report the number of motile spermatozoa recovered, but they obtained embryos by IVF using swim-up in alpaca epididymal sperm.

The second main method used, discontinuous density gradient centrifugation, is not only used to separate spermatozoa, but also many different types of cells (76). In this technique, the selection is based on the fact that only highly motile spermatozoa can go through different density gradients and reach the bottom of the tube. It requires layering a high-density medium over a lower density medium in a tube. Spermatozoa are then placed over the layers and are centrifuged, moving the cells through the gradient until they reach an area where their density is similar to that of the medium. Spermatozoa can be separated from other elements present in the ejaculate (such as debris, bacteria, epithelial cells, and leukocytes) due to their different density; thus, after centrifugation, seminal plasma remains at the top of the column and sperm form a pellet at the bottom (76). Motile sperm form this pellet faster than immotile spermatozoa because they line up with the direction of the centrifugal force and hence, if centrifugation time and force are selected with care, motile sperm can be successfully isolated (76). The main advantage of this technique is that it can be used with ejaculates with low sperm concentration. Moreover, the fraction of sperm that is obtained is motile, clean, and has reduced ROS. The disadvantages lie in its cost and in the difficulty in preparing the gradients.

Because one of the SAC semen characteristics is a low concentration, in these species, density gradient centrifugation would be the best choice to select spermatozoa. There are two studies reporting the use of density gradient centrifugation in llamas to produce embryos by ART: one using ISolate^®^ (Irvine Scientific, Santa Ana, CA, USA) (38) and the other using Percoll^®^ (39). Sansinena et al. (38) layered 0.5 ml extended semen over a discontinuous gradient system in HEPES-buffered human tubal fluid (50–90%; ISolate^®^) centrifuging at 400 *g* for 15 min. The authors did not report the number of motile spermatozoa recovered, but they obtained embryos by ICSI using this technique on semen obtained by aspiration from the anterior vagina. In our laboratory, embryos have been produced both by IVF and ICSI using ejaculates with reduced thread formation after incubation in 0.1% collagenase in TALP (39). In this study, after collagenase treatment and removal of the enzyme, the pellet was placed over a Percoll^®^ column (45% density). The sample had a concentration of 19.2 ± 17.1 × 10^6^ spermatozoa/ml, a progressive motility of 56.36 ± 17.47%, a percentage of live sperm cells of 58.5 ± 15.91% (6-carboxifluorescein diacetate and PI stain: CFDA/PI) and a percentage of spermatozoa with functional membranes of 53.35 ± 19.76% (HOS test). “As the use of Percoll^®^ for density gradient centrifugation presents the risk of contamination with endotoxins (83), washing selected sperm is recommended. This technique requires a high number of centrifugations to wash cells of possible toxins, which can induce damage to the sperm by an increase in ROS production (86) and involves a longer treatment period” (83). For all the reasons referred to above, our laboratory evaluated and put into practice a protocol using Androcoll-E™, obtaining llama embryos by IVF after using this method of sperm selection (15, 22, 87). Androcoll-E™ is a single layer centrifugation variation of the previously mentioned sperm separation technique, where instead of a density gradient, only one layer of colloid is used (76). It has been reported that Androcoll-E™ not only selects motile sperm but also morphologically normal sperm with intact DNA (88). In llamas, the protocol included incubating the ejaculates in a solution of 0.1% collagenase in H-TALP-BSA followed by centrifugation during 8 min at 800 *g* to decrease seminal plasma thread formation. “The pellet was re-diluted in 2 ml of H-TALP-BSA and placed over 2 ml of Androcoll-E™ and, following centrifugation at 600 *g* for 20 min, this second pellet was re-diluted in 0.5 ml of Fertil-TALP and sperm concentration, motility, and viability (using CFDA/PI) were evaluated” (22). Using this technique, the pellets obtained contained 10–40 × 10^6^ spermatozoa/ml, with a progressive motility that varied between 20 and 60% and a viability of 20–80% (89).

Recently, our laboratory compared Percoll^®^ and Androcoll-E™ to select raw llama semen incubated with collagenase observing no significant differences in morphologically normal or in viable sperm between raw semen and both selection techniques. However, only sperm recovered from Androcoll-E™ did not show a significant increase of sperm with bent tails (85). Percoll^®^ has also been used to select alpaca epididymal spermatozoa (12). These authors report embryos produced by IVF using this method of sperm selection, but they did not mention the quality of the samples selected.

## *In Vivo* Embryo Production

*In vivo* embryo production can be carried out from a dominant follicle present in the ovary of the donor female (single-ovulation protocol) or by stimulating the growth of multiple follicles in donor females to increase the number of embryos recovered. Applying exogenous hormones for ovarian superstimulation benefits the intensive use of genetically selected females.

### Management of Embryo Donor Females

Embryos from a single dominant follicle can be obtained between 6 and 8 days after mating (day 0). According to Sumar (90), alpaca embryo collection rates from the uterus are optimal as from 7.5 days after mating, but are low at day 6 and inconsistent between days 6.5 and 7. This author has also reported that it is feasible to expect an embryo from 85% of the flushes carried out in donors subjected to a single-ovulation protocol and induced to ovulate every 12–14 days (90). Egey et al. (91) worked during 60 days with nine embryo donor llamas. They controlled the ovarian activity by daily transrectal ultrasonography until the presence of a dominant follicle and females were then mated. During this period, 5 uterine flushings were made per female (45 in total) and 24 embryos were obtained with a recovery embryo rate of 53.3% (24/45), 85% of these were from successive follicular waves. Similar to Sumar’s work (90), Egey et al. (91) obtained a mean interval between uterine flushings of 13.16 ± 3.12 days (mean ± SD).

Working with superstimulated embryo donor females, the protocols for follicular inhibition are the same as we described before. With regard to superstimulatory treatment, our team uses doses of 1,000 IU of eCG to obtain various embryos from the same donor llama. We don’t use the higher doses, to avoid the possible displacement of the ovarian bursa due to an exaggerated ovarian response. Using this protocol, a total of 67 embryos were collected from 22 donor females (3.04 ± 0.11 embryos/female, mean ± SE) (92) whereas Carretero et al. (31) obtained 74 llama embryos after flushing 28 donors (average 2.6 embryos/flush). In a retrospective study in alpacas, after the administration of an FSH superstimulation protocol, a total of 4,188 embryos were collected during transcervical uterine flushing from 1,636 animals (2.57 ± 3.01 embryos/female) (93).

#### Induction of Ovulation

With the objective of obtaining a high percentage of ovulations after superstimulatory treatments, the embryo donor females are monitored ultrasonographically and are mated when they present two or more dominant follicles (≥7 mm). The period between the day the superstimulatory treatment was administered and detection of dominant follicles was 5 days (14). The number of matings per donor female varies, but in our program, we carry out two matings separated by an interval of 24 h to increase the number of available spermatozoa and using two different males of proven fertility to minimize the male effect. Although ovulation occurs in response to the matings (94), because there are so many follicles, the female donors receive a single IV dose of 8 µg of buserelin after the first copulation, to maximize the ovulatory response of the ovary.

Ovulation of the donor is detected by ultrasonography or progesterone assays. Visualization of the CL is usually difficult in the first 3 days post-ovulation but becomes easy thereafter. Plasma progesterone levels start to increase 4 days after mating, reach high levels (12 nmol/l) by day 8, and then start to decline and reach basal levels by days 10–11 (3).

### Embryo Recovery

Non-surgical recovery of *in vivo* produced embryos is carried out by transcervical uterine flushing. Ovulation occurs in llamas 28.6 ± 0.36 h after buserelin administration (95) and the embryo reaches the uterus approximately 6 days after ovulation (96). Due to this, embryo recovery is carried out using uterine flushing between 7 and 8 days after mating. Working with superstimulated females, we prefer to flush on day 8, to increase the possibility of obtaining all the embryos that developed, including those produced by follicles that ovulated later.

#### Uterine Flushing Technique

**Figure 2:**
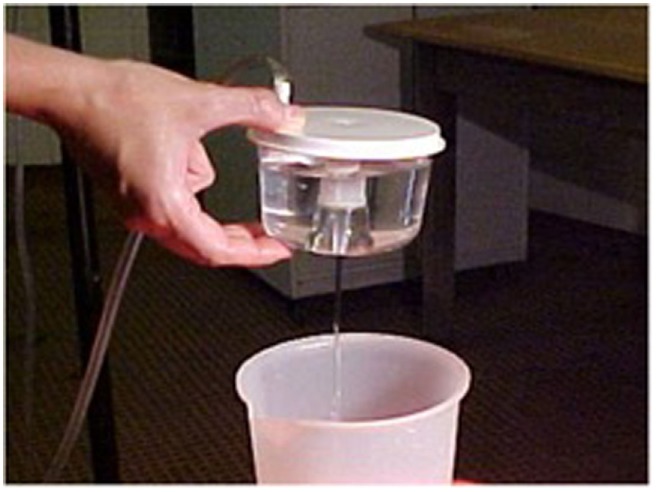
Uterine flushing media recovered through a VCI^®^ High Volume filter for embryos into a graduated container.

The maneuver can be carried out with the female in sternal recumbency or standing up, but restrained in stocks. After wrapping the tail, the rectum is emptied of feces and the perineum should be washed with a weak solution of iodine, rinsed carefully with clean water, and then dried. If necessary, 0.2 mg/kg xylazine IV (Rompun^®^, Bayer, Buenos Aires, Argentina) can be administered to belligerent females before the flushing procedure. Some authors suggest the use of epidural anesthesia but we don’t consider it necessary, especially if they are already sedated. “Collection is conducted using a Foley catheter (12 or 16 Fr, according to female size) and a stylet is inserted into the catheter to keep it from bending during rectovaginal manipulation” (63, 87, 92). The catheter is inserted into the vagina and the cervix is threaded with manual help through the rectum, until the catheter reaches the uterus. After placing the catheter cuff immediately cranial to the internal cervical *os*, it is inflated with air according to the catheter gage (5–10 ml) prior to conducting the uterine flushing proper (63, 87, 92). The uterus is flushed 4 to 5 times with Ringer Lactate solution, using a total volume of 500 ml. As the fluid enters the uterus, the complete filling of both uterine horns must be controlled by rectal palpation, until the uterus walls become tense, and at that moment, the valve controlling the exit of the fluid is opened. After passing through a 65 μm VCI^®^ High Volume filter for embryos, the fluid is collected in a graduated container to evaluate the quantity of media recovered (Figure 2). All materials coming into contact with the embryos, such as the flushing fluids and the filter, must be kept between 37 and 38°C to avoid damaging the embryos. During each flushing, the uterus must be massaged through the rectum to aid total recovery of the fluids and keep the embryos suspended in the media. More than 90% of the fluids must be recovered, free from cell debris and blood. A single IM dose of 250 µg of cloprostenol is administered to the donor females immediately after the flushing to produce lysis of all CLs in both ovaries.

### Embryo Management and Evaluation

**Figure 3:**
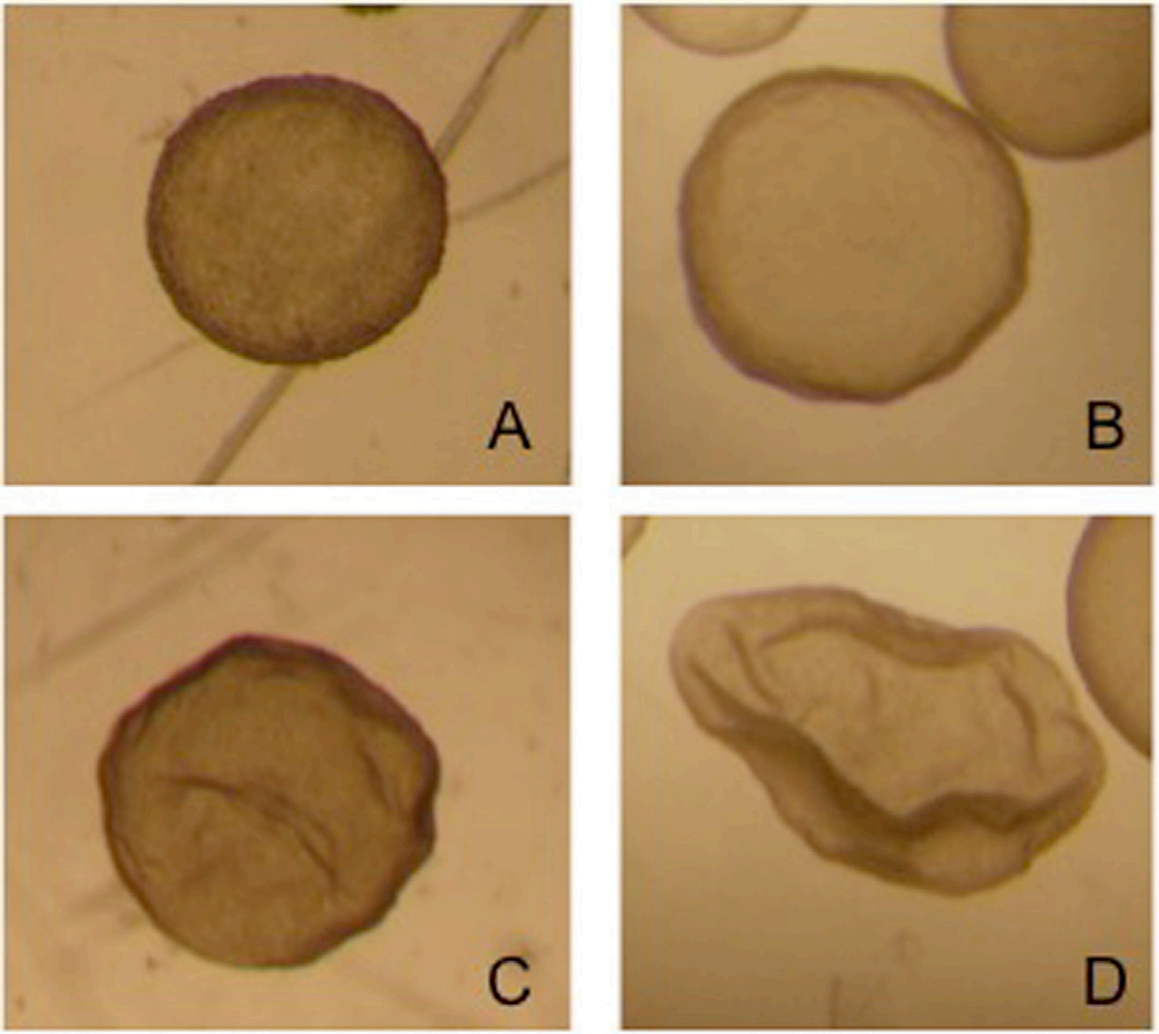
Four different grade 1 llama embryos: **(A)** spherical and symmetrical embryo, **(B)** spherical embryo with a slightly irregular contour, **(C,D)** collapsed embryos, starting the elongation process.

The contents of the filter are emptied into a searching grid dish, on a warm stage, and under laminar flow. The filter is rinsed with flushing medium to ensure that the embryo is not lost in the filter. The dish is searched under a stereoscope (40×). All embryos recovered on day 8 can be seen with the naked eye. Once the embryos have been identified, they are washed four times by successive transfers to 1 ml drops of Ringer Lactate solution and are classified according to their morphological characteristics and stage of development into five grades, as reported by Tibary and Anouassi (97). Some authors (90, 98) use the International Embryo Transfer Society (IETS) bovine embryo classification, but we consider that dromedary classification is more appropriate because of the similarity of the blastocysts (Figure 3).

**Figure 4:**
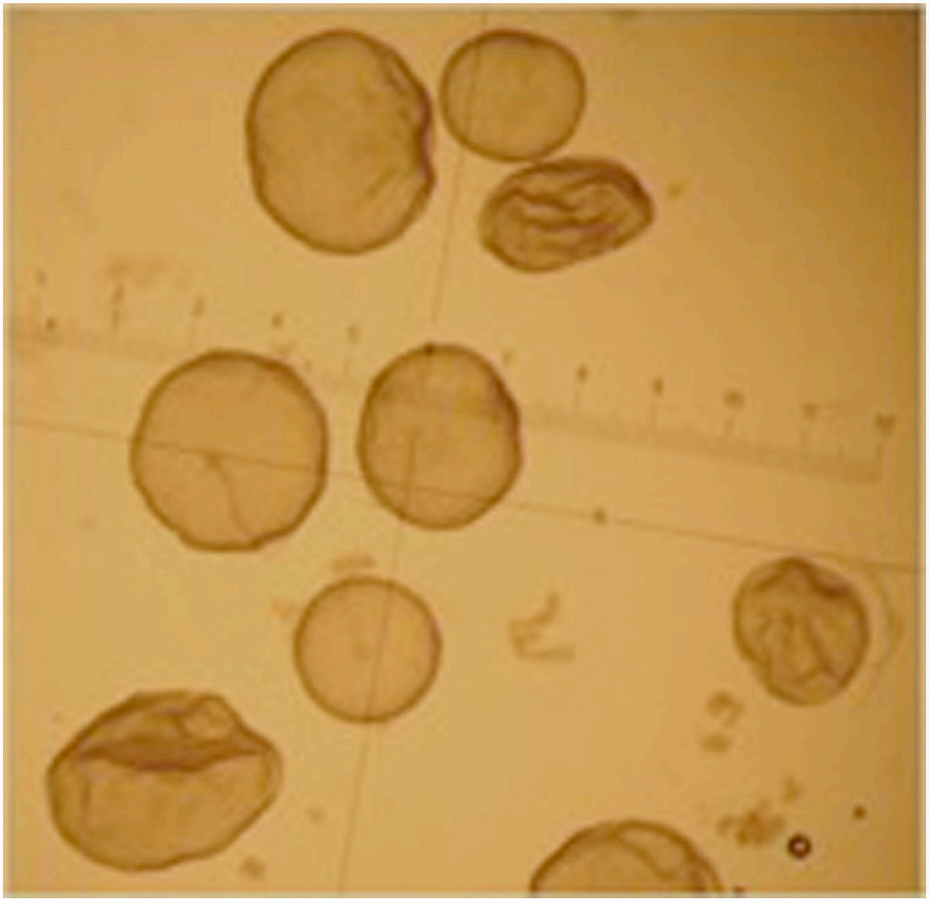
Different grade 1 embryos obtained from the same superstimulated female on day 8 after mating.

Embryos recovered from the uterus in llamas are generally at the hatched blastocyst stage and the size of the embryo is highly variable: from 0.3 mm to 1.2 mm on day 8 after mating (92). This variability in size is due to the different ovulation time of each follicle in superstimulated females. The embryos produced by the first ovulations lose their spherical shape and at the time of recovery appear collapsed, possibly already starting the elongation process (Figure 4).

According to Vaughan et al. (93), the diameter of alpaca embryos collected from the single ovulation group was analogous to that of the multiple-ovulation embryo transfers group (1.29 ± 0.76 vs. 1.41 ± 0.75 mm, respectively; *p* > 0.05).

## Embryo Preservation

Cryopreservation is a technique that permits the storage and transport of cells (somatic, gametes, or embryos) from genetically superior animals, maintaining their viability for an unlimited length of time, so they can be used in the future. Advances in the cattle industry have allowed embryos to be frozen with little decrease in fertility upon thawing and transfer. Limited studies have been conducted on cryopreservation of SAC embryos and no live offspring have been reported to date (99).

### Deep Freezing and Vitrification

There are two methods of cryopreservation: the conventional method, which consists in a slow temperature descent and requires adequate equipment; and vitrification, which consists in rapid cooling and does not require equipment for the temperature descent. Damage to the cells can occur in both methods, but the type and degree of damage probably depends on the method used (100). In llama, the feasibility of cryopreserving embryos using both methods was compared. Similar percentages of survival were obtained (54% with vitrification and 57% with slow cooling) but vitrification is a more attractive technique for applying in the field because of its simplicity (101). The advantages of vitrification include: rapid temperature curves, use of very small volumes and a lower probability of cell damage related to intra and extracellular ice formation. In llama, vitrified embryos have been successfully transferred, resulting in a 50% pregnancy rate (2/4; two embryos per recipient) (102). One of the reasons embryo cryopreservation is difficult in llamas is the very large size they present at the time of recovery from the uterus [from 0.3 mm to 1.2 mm on day 8 after mating; (92)]. All embryos recovered non-surgically on day 7 or 8 after the first mating were at the hatched expanded blastocyst stage (92, 103). Collection of embryos at an earlier stage of development could improve the results in terms of post-thaw viability. For example, in llamas, Miragaya et al. (64) used retrograde oviductal flushing to surgically recover morula-stage embryos.

Another reason for the disappointing results in embryo cryopreservation is the great amount of lipids in embryos. Similar to porcine embryos, camelid embryos contain a high concentration of lipids; this has been shown to have an adverse effect on the conventional freezing methods (99). von Baer and Del Campo (104) vitrified 0.3–0.8 mm llama embryos by placing them directly in 40% ethylene glycol, in one step, before immersing them in liquid nitrogen using open pull straws as the support system and then evaluated the results in terms of hatched blastocyst morphology and survival. Acceptable re-expansion was observed after thawing; however, pregnancy rate was 0% (104). In an effort to reduce the exposure time to toxic cryoprotectants, a microinjection device has also been designed to allow the injection of a cryoprotectant solution directly into the cavity of hatched llama blastocysts and has resulted in a 67% (2/3) pregnancy rate (105). More research is needed to determine the best method for llama embryo cryopreservation.

## Embryo Transfer

Embryo transfer is one of the most widespread biotechnologies in domestic animals. In addition to shortening the generation interval, it allows a large number of offspring of genetically superior animals to be obtained.

At the time of ET, there are various factors to be considered, one of them being embryo morphology. As previously mentioned, embryos are classified into five grades according to their morphological characteristics and stage of development (97). We only transfer grades I and II, where grade I are embryos of excellent quality, with a smooth surface and with a size that corresponds to the day of collection; and grade II are good embryos, similar to those of grade I, but with a few irregularities of their contour and not many protruded cells.

Selection of the recipient females is one of the most important factors for ET to be successful. Our selection criteria are based fundamentally on: good body conformation, age (young females, between 4 and 8 years of age), optimum health, ultrasound evaluation of ovarian activity, and absence of fluid in the uterus.

### Synchronization with Donors

Another important factor is the day the ET is carried out after ovulation. Exposure of the embryo to a uterus that is asynchronic regarding its stage of development can bring about an untimely presence of proteins, growth factors, and hormones that can lead to retarded embryo development or even death. In camelids, the CL half-life is very short, only 8–10 days [llama: (3); dromedary: (106)], this indicates that the time frame in which to transfer the embryos to the uterus, before luteolysis occurs, is short. It is yet unclear as to the optimum degree of synchrony needed between donors and recipients that will achieve the highest embryo survival rates in SAC and hence there are no reports on this aspect. ET results suggest that the best recipient should have ovulated on the same day as the donor or up to 48 h after (90, 92). Nevertheless, Vaughan et al. reported in 2003 in alpacas that 7 or 8 days after GnRH analog administration were the most advantageous for transfer into recipients. Our team carries out ET to recipient females on day 6 after induction of ovulation because the endogenous prostaglandin levels are still basal at this time, only beginning to rise on day 7 (1).

### Transfer of Embryos

The ET technique can be carried out either surgically or non-surgically. Sumar and Franco reported the first alpaca born from a surgical ET in 1974 (107). “In llamas, the first birth obtained from a non-surgical ET was reported by Wiepz and Chapman (108) and the first birth obtained using this technique in superstimulated donor females was in 1992, reported by Bourke et al. (27, 109).” This procedure has become widespread as, in domestic species, such as bovines and equines, it is a very useful production method. In camels, ET was initiated to respond to a huge demand from the camel industry particularly in the United Arab Emirates since 1990 (110). However, the technology still plays a minor role in SAC (93, 102, 109, 111). One of the reasons for the slow progress in this species could be, as we already mentioned, due to the release of PGF_2α_ for the CL luteolysis, which begins on day 7 or 8 post-mating and is completed 1 or 2 days later. This event reduces the CL half-life to 8 or 9 days (1, 3), thus restricting the period for effective transfer of embryos to the uterus and for MRP to successfully maintain the CL viable (92). Additionally, it is usually thought that the poor pregnancy rates obtained after transcervical ET are due to cervical manipulation inducing a large release of prostaglandin, a weak signal from the embryo during MRP or to insufficient or inadequate embryo migration (92). In other species, such as the mare, cervical manipulation induces luteolysis (112) and yet, a study by Trasorras et al. (92) in llamas, showed that threading of the cervix during the ET procedure was not accompanied by a decrease in plasma progesterone concentrations. It has been observed in dromedaries that the passage of the ET pipette through the cervix produces PGF_2α_ release from the endometrium, but not in enough quantity to induce luteolysis (113). Likewise, in llamas, we have observed that “cervical threading together with transfer of sterile PBS to simulate the complete ET maneuver does not alter plasma progesterone concentrations” (92), with the relationship between the place that PBS is deposited and CL localization not being important (92).

More than 99% of pregnancies in SAC are carried out in the left uterine horn despite ovulations occurring with equal frequency in either ovary (4, 5, 114). “The luteolytic action of the right uterine horn is solely local, while the luteolytic action of the left uterine horn is on the CL located on the left and right ovaries” (2, 92). Pregnancy loss is high in these species, with over 50% being registered during the first month of gestation, and the reasons for this are still unknown (115). However, when taking into account the first 90 days of pregnancy, this loss rises to 60–80% (116). This difference in the luteolytic action of endometrial prostaglandin in both uterine horns together with the left horn gestations and the high pregnancy losses, lead one to think that the right horn is incapable of carrying a pregnancy to term and, therefore, that “the success of ET could perhaps depend on the site where the embryo is deposited in the uterus” (92). This was supported by data in the study that showed the highest ET results (50%) in llamas were attained when the embryos were placed in the left uterine horn with a CL in the ipsilateral ovary (92). Sumar and Leyva hypothesized in 1979 that when ovulation occurs in the right ovary, for the embryo to perform MRP, it must travel from the right uterine horn to the left (117). “In alpacas, ET rate was significantly higher in recipients in which the embryo was transferred into the left uterine horn when the CL was on ipsilateral ovary (2,241 transferred embryos, 963 crias born, 43%) in comparison to recipients that had the CL on the contralateral ovary (1,976 transferred embryos, 795 crias born, 40.2%)” (93). Therefore, studies in ET would seem to agree that in order to improve pregnancy rates with this technique, it would seem that the most desirable option would be to place the embryo in the left uterine horn with an ipsilateral CL (92, 93).

## Concluding Remarks

A lot of issues surrounding the production of SAC embryos still needs to be enhanced. Due to the lack of information concerning basic reproductive characteristics of SAC, for numerous years, many of the protocols applied in camelids were based on knowledge borrowed from and successfully applied to other species. Today, despite advances in research in complex technologies such as nuclear transfer, there is still much to be learnt regarding these species, both in the female and in the male physiology. Among the physiological characteristics that need further research, our opinion is that the most important are: ovarian dynamics and management of the follicular waves, oocyte maturation *in vivo*, gamete interaction in the oviduct and the influence of the oviductal environment on the sperm reservoir and on early embryo development (especially with regard to requirements for early embryo growth), dynamics and mechanisms of MRP, effect of seminal plasma on both gametes and on the mechanism of ovulation in this species.

Notwithstanding, other ART such as ET, present encouraging results and can be carried out with promising success.

We have described the different assisted reproduction techniques currently applied in SAC, and it becomes evident that some factors need to be improved, among them, the most urgent are:

– Follicular inhibition and ovary superstimulatory protocols.
– *In vitro* oocyte maturation.
– Gamete cryopreservation.
– Timing and doses for AI.

The commercial application of *in vivo* or *in vitro* embryo production and preservation in camelids will depend on the successful development of these points.

## Author Contributions

VT wrote female sections. MC and SG wrote male sections of the review. DN, MC, and MM provided significant advice on the document structure, content, and editing as this document evolved to its current form. All the authors read, edited, and approved the final manuscript.

## Conflict of Interest Statement

This review was conducted in the absence of any commercial or financial relationships that could be construed as a potential conflict of interest.
